# Intensification of Nickel Bioleaching with Neutrophilic Bacteria *Guyparkeria halophila* as an Approach to Limitation of Sulfuric Acid Pollution

**DOI:** 10.3390/microorganisms9122461

**Published:** 2021-11-29

**Authors:** Tatiana Abashina, Alyona Yachkula, Elena Kaparullina, Mikhail Vainshtein

**Affiliations:** Federal Research Center “Pushchino Scientific Center for Biological Research of the Russian Academy of Sciences”, Skryabin Institute of Biochemistry and Physiology of Microorganisms, Microorganisms Russian Academy of Sciences, Prospect Nauki 5, 142290 Pushchino, Russia; tnabashina@gmail.com (T.A.); repinaalenk@mail.ru (A.Y.); lenokap80@gmail.com (E.K.)

**Keywords:** nickel bioleaching, low-grade ore, formic acid, *Guyparkeria halophila*

## Abstract

Hydrometallurgical production of valuable and non-ferrous metals is traditionally accompanied with acid waste effluents/acid mine drainage leading to acidification of the mining areas. The traditional cause of this pollution is the well-known technology based on the recovery of metals with acid solutions and the application of strong acidophilic leaching bacteria for the oxidation of sulfide ores. In our experiments, we used neutrophilic autotrophic bacteria (NAB) stimulated with formic acid or coupled with acidophilic bacteria. The first approach was based on using formic acid as an energetic substrate by autotrophic bacteria. In the second case, the NAB provided initial biogenic acidification for the following growth of the inoculated acidophilic bacteria. Our experiments resulted in increased nickel recovery from the low-grade sulfide ores, which was provided by the NAB in a medium supplemented with formic acid. Bioleaching resulted in 1116 mg Ni/L (69.75%) in the medium with formate and only 35.4 mg Ni/L without formate in 43 days. As a whole, our bench scale experiments showed that the stimulated NAB can be effective at pH 7–5. Partially replacing sulfuric acid with formic acid could also give benefits via the following natural degradation of acid wastes. As a whole, this approach is more environmentally friendly than conventional bioleaching techniques.

## 1. Introduction

Hydrometallurgical mining processing produces acid waste effluents/acid mine drainage. In turn, these effluents provoke a large-scale technogenic catastrophe: acidification of the mining areas with strong inorganic acids, usually-with sulfuric acid [1,2]. The traditional technique for metal recovery from the low-grade sulfide ores was chemical leaching with strong acids; since the 1950s [3], this chemical leaching has been partly replaced with bioleaching, i.e., the application of acid-producing leaching microorganisms. The leading industrial species of these microorganisms are presented with acidophilic autotrophic bacteria or archaea. The choice was provided by the following characteristics of industrial significance: (i) strong acidophilic bacteria produce sulfuric acid by the oxidation of mineral sulfides and/or increase redox values by iron oxidation and (ii) bacterial autotrophy excludes any need to support the industrial process with organic growth substrates. During bioleaching of sulfide ores, strong acidophilic bacteria oxidize mineral sulfides to sulfuric acid, with an essential pH decrease and environment acidification. Attempts to expand the diversity of industrial leaching moderately acidophilic bacteria were not successful enough to replace strong acidophilic species such as *Acidithiobacillus ferrooxidans*, which can oxidize both mineral sulfides and Fe^2+^, grow at pH < 3.0, and synthesize its biomass from carbon dioxide [4,5].

For decades, microbiological renovations were focused on (i) the search for more effective acidophilic species and strains [6,7,8] or (ii) optimization of the processing regimes [9,10,11]. Good examples of these approaches are presented in some modern research investigations where authors used (i) a new *Sulfobacillus* strain, (ii) a higher temperature (47 °C), and (iii) low pH (1.8) [12]. Some approaches also included combinations of different microorganisms such as acidophilic chemolithotrophic and heterotrophic strains (*Sulfobacillus thermosulfidooxidans* and *Thermoplasma acidophilum*) [13].

Three decades ago, Pronk with coauthors [14] had already shown that the growth of *A. ferrooxidans* (former name *Thiobacillus ferrooxidans*) can be stimulated with formic acid. By the published data [14], formic acid, as an organic compound, was not used as a growth substrate but it provided some energetic needs. Moreover, the mentioned authors also proposed pre-growing *A. ferrooxidans* with formate for the following metal leaching from ores and claimed this approach with a patent [15]. This increase in the activity of acidobacilli with formate served as the starting point for the presented study.

Sulfuric acid supplementation is essential to provide low pH values for strictly acidophilic leaching bacteria (pH optimum 1–2). In this regard, we studied the stimulation of neutrophilic autotrophic bacteria (NAB). The studied bacteria, *Guyparkeria halophila* VKM B-2757D, are known as NAB that oxidize reduced sulfur compounds, including sulfide, to sulfuric acid at pH optimum 7.0–7.3 [16]. Additionally, this species is moderately halophilic, which is favorable since the process increases total mineralization of the leaching solution. Our data on nickel bioleaching from the low-grade sulfide ores showed an intensification of nickel recovery by the NAB despite the limited input of sulfuric acid. The developed approach seems to be a kind of eco-friendly technology because it both decreases the input of sulfuric acid and simplifies the following acid waste disposal.

## 2. Materials and Methods

### 2.1. Mineral Materials

Samples of the low-grade sulfide nickel ore, 0.8% Ni, were presented by CANMET-Mining and Mineral Sciences Laboratories. Mineral composition of the ore is presented in the Table 1 for the most essential compounds. Mechanical composition of the samples was represented by a fine (<1 mm) fraction.

### 2.2. Microorganisms and Culturing Media

Two bacterial strains were used in the leaching experiments, namely: *Guyparkeria halophila* VKM B-2757D and *Acidithiobacillus* sp. KZ-02. The last of these was originated from the acid mine drainage, ore deposit Aksu (isolated by I. Korovkina, “National Center for Biotechnology of the Republic of Kazakhstan”).

Neutrophilic strain *G. halophila* was cultured in the medium DSM 518 (g/L): Na_2_HPO_4_·2H_2_O, 7.9; KH_2_PO_4_, 1.5; NaCl, 50; MgSO_4_, 0.1; NH_4_Cl, 0.4; Na_2_S_2_O_3_ (anhydrate), 5.0; trace element solution, 5.0 mL; pH was adjusted to 7.0. The 10% solutions of MgSO_4_·7H_2_O, NH_4_Cl, CaCl_2_·2H_2_O, and 25% Na_2_S_2_O_3_ were sterilized separately and added to the main volume of the medium after sterilization. pH 7.0 was chosen in accordance with the species characteristics [16].

Acidophilic strain *Acidithiobacillus* sp. KZ-02 (pH optimum 4.5) was cultured in synthetic mineral medium of the following composition (g/L): KH_2_PO_4_-0.5; NH_4_Cl-0.5; MgSO_4_·7H_2_O-0.25; Na_2_S_2_O_3_·5H_2_O-5.0; NaCl-0.5. The medium pH was 5.0. To adjust pH to the desired value, H_2_SO_4_ was used.

### 2.3. Leaching Experiments

In experiments with the NAB culture *G. halophila*, 40 g dry ore samples were placed into 750 mL flasks with 200 mL leaching solution, i.e., the solid and liquid phases were at a ratio of 1:5. As mentioned above, mechanical composition of the samples was represented by a fine (<1 mm) fraction and the nickel content was 0.8%. Theoretical maximum amount of nickel in solution could reach 1600 mg/L. All experiments were conducted in triplicate under aerobic conditions at 28 °C on a shaker IBPM (IBPM, Pushchino, Russia) at 180 rpm. Thus, the process was a model of vat leaching at constant temperature. These experiments included comparison of nickel leaching in the culturing medium DSM 518 with formate (0.3%) and without formate (blank). Inoculate was 10 mL of this mineral medium with 10^6^–10^7^ cells/mL.

In two-step experiments combining NAB and acidophilic bacteria, at the first step, the flask with ore was inoculated with *G. halophila* culture. The second step of the experiment: when pH decreased from 7 to 5, the flask was additionally inoculated with *Acidithiobacillus* sp. KZ-02. Experimental conditions were the same as described above.

The control (blank) experiments were presented as versions without inoculation.

### 2.4. Nickel Analyzes

The aim of the study was the leaching of nickel, since its extraction was of not only scientific but also industrial interest. We did not assess leaching of associated metals. Nickel concentrations in solution were analyzed with ion chromatography. The ion chromatography analyses were performed with device Compact IC, columns 6.1010.300 Metrosep: 3 × 9 × 150 mm (Metrohm, Herisau, Switzerland).

### 2.5. Enzyme Activity Analyzes

Bacterial cells were harvested at the beginning of the stationary growth stage and precipitated by centrifugation at 6000 *g* for 15 min; then they were washed with buffer solution and re-suspended. At the next step, the cells were disrupted by a Qsonica S-4000 sonicator (Qsonica LLC, Newtown, CT, USA) in tubes placed in ice. The disruption procedure was repeated 6 times, 30 s each, at 1 min intervals. Unbroken cells were separated by centrifugation at 14,000 *g* for 40 min. The resultant supernatant was used as a target extract to analyze the enzyme activity. Formate dehydrogenase was estimated by the reduction of 2,6-dichlorophenolindophenol (DCPIP) [13]. The reaction mixture contained (μM): phosphate buffer (pH 7.0), 50.0; DCPIP, 0.075; phenazine methosulfate (PMS), 0.5; formate as substrate, 50.0; cell extract. NAD^+^-dependent formate dehydrogenase was estimated by reduction of NAD^+^ [17]. The reaction mixture contained (μM): tris-HCl buffer (pH 7.5), 50.0; NAD, 0.25; formate as substrate, 50; cell extract. The reactions were started by addition of formate. The protein content was determined by a modified Lowry method [18].

### 2.6. Optical Density Analyses

Optical density (OD) of the culture was measured using Spekol 221 spectrophotometer (Carl Zeiss Industrielle Messtechnik GmbH, Jena, Germany) at wavelength 600 nm. The data are the mean values of triplicate experiments and duplicate determinations, variations were within the limits of 5%.

### 2.7. Statistics

The experiments and analyses were performed in triplicate. All data were statistically checked and the presented results showed a high level of reliability: *p* > 0.99 (ANOVA, Excel Microsoft). Error bars are shown in the Figures.

## 3. Results and Discussion

### 3.1. Stimulation of Bacterial Growth with Formate

**Figure 1:**
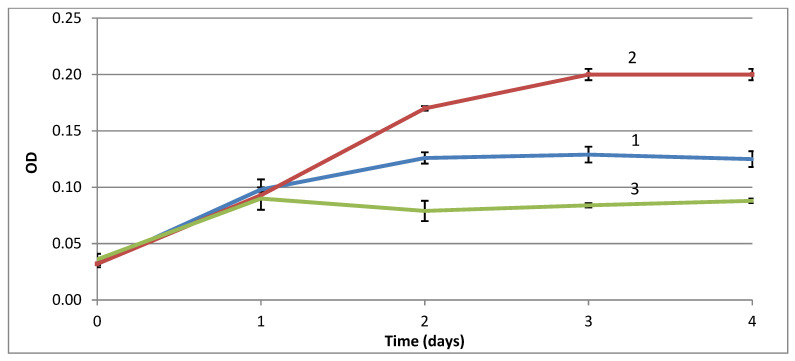
Effects of formate on growth dynamics of *G. halophila* measured by optical density in the medium DSM 518. Abscissa axis: time (days); ordinate axis: optical density (OD), λ = 600 nm. Formate concentrations, %: 1–0.0; 2–0.3; 3–0.6. Error bars are shown in the Figure.

It is already known that autotrophic *A. ferrooxidans* ATCC 21834 (former name *Thiobacillus ferrooxidans*) uses organic compound, formate, as a source of energy [14,15]. This way of formate consumption was suggested by Pronk with coauthors [14] because the studied bacteria were stimulated by formate and contained phenazine methosulfate (PMS) formate dehydrogenase. We investigated if moderate acidophilic autotrophic bacteria *G. halophila* VKM B-2757D could also be stimulated with formate. The experiments showed stimulation of the bacterial growth with 0.3% formate in the DSM 518 medium and the growth depression with 0.6% (Figure 1). Enzyme activity of the formate dehydrogenase was investigated and compared with the published data on *A. ferrooxidans* ATCC 21834 (Table 2).

Thus, following data published by Pronk with coauthors [14], we also may conclude that bacteria *G. halophila* oxidize formate but do not use it for direct biomass synthesis. It is interesting to note, however, that the initial composition of the medium does not include carbonate, i.e., carbon as a growth substrate is limited with dissolution of CO_2_ in the acid culturing medium. At the same time, the oxidation of formate to carbon dioxide not only supplies the bacteria with energy but also provides an increased concentration of CO_2_ directly on the cell surface. We can assume that formate is not only an energy substrate but also a precursor of a growth substrate.

### 3.2. Stimulation of Ni Leaching by G. halophila with Formic Acid

**Figure 2:**
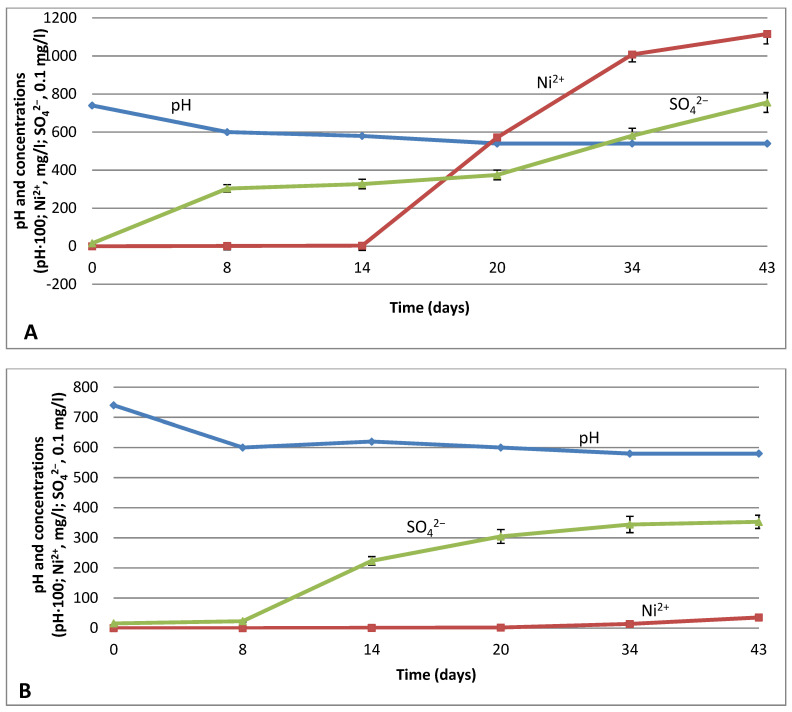
Effects of formate supplementation on bioleaching of sulfide nickel ore inoculated with *G. halophila*. (**A**) medium DSM 518 supplemented with formate, 0.3%. (**B**) medium DSM 518 without formate (blank). Abscissa axis: time (days); ordinate axis: concentrations and pH (Ni^2+^, mg/L; SO_4_^2−^, mg/L × 0.1; pH × 100). Error bars are shown in the Figure (some error values are too small to be distinctive).

The whole study was oriented to limitation of the sulfuric acid input for the metal leaching process. Our main approach was based on the application of NAB, which grows at neutral or moderate low pH. As was shown above, stimulation with 0.3% formate increased growth 2.5-fold in 4 days (Figure 1). These data were a logical basis for the nickel leaching from low-grade sulfide ore using formate-stimulated NAB. The results of the experiments are presented in Figure 2. The medium pH decreased together with bacterial sulfate production, and in 14 days, pH values reached 5.8 and 6.2 in variants with and without formate, respectively. Sulfate production reached 3271 ± 25 and 2235 ± 14 mg SO_4_^2−^/L, respectively, in 14 days and increased up to 7557 ± 52 and 3532 ± 22 mg SO_4_^2−^/L in 43 days. Similarly, concentrations of dissolved nickel were 3.10 ± 0.4 and 0.86 ± 0.07 mg/L, respectively, in 14 days. Prolonged processing resulted in 1116 ± 8 mg Ni/L in the medium with formate and only 35.4 ± 0.5 mg Ni/L without formate in 43 days (Figure 2). The presented result of the maximum nickel yield is equal to 69.75% of the full nickel content. It is interesting to compare our results with the data on bioleaching by strictly acidophilic *A. ferrooxidans* without formate. A good example is presented by Yanishevskya with coauthors [19] where nickel yield from sulfide ores was 22.5%. This result is 10 times higher than our nickel yield without formate (2.21%), but three times lower than our yield with formate.

When the initial pH value was neutral (pH 7.0–7.5), no changes in nickel and sulfate concentrations, or in pH, were discovered in the uninoculated (blank) versions during exposition.

### 3.3. Subsequent Leaching with Neutrophilic Bacteria G. halophila and Acidophilic Bacteria Acidithiobacillus sp.

Traditional metal leaching is carried out by the acidic degradation of minerals and solution of metals in the acid leaching solution. Thus, strong acidophilic chemolithotrophic bacteria leach metals from sulfide ores via the oxidation of sulfides to sulfuric acid. As far as industrial leaching strains need low initial pH, the processing is provided with the supplementing of strong acids (sulfuric acid).

**Figure 3:**
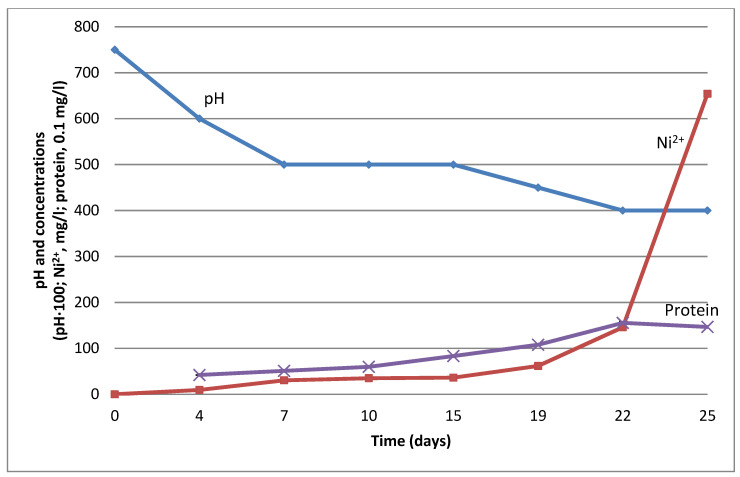
Two-step Ni bioleaching from the low-grade sulfide ore. The sample was initially inoculated with *G. halophila* VKM B-2757D and additionally inoculated with *Acidithiobacillus* sp. KZ-02 (at the 15th day, shown in the figure with arrow). Abscissa axis: time (days); ordinate axis: concentrations and pH (Ni^2+^, mg/L; protein, mg/L; pH × 100). Error bars in the Figure are too small to be distinctive.

The isolated strain *Acidithiobacillus* sp. KZ-02 was acidophilic and did not grow at pH above 5.0. Initial medium pH was 7.5, so, we inoculated the medium with NAB, waited till they decreased pH to 5.0 and then inoculated it additionally with *Acidithiobacillus* sp. KZ-02. The data of the experiment are shown in Figure 3.

Ore inoculation with the NAB *G. halophila* VKM B-2757D resulted in the pH decreasing from 7.5 to a stable 5.0 in ten days. The process of Ni leaching also came to a plateau in ten days. At the 15th day, the following inoculation with *Acidithiobacillus* sp. KZ-02 resulted in the next step of pH decreasing and additional Ni leaching (Figure 3).

Figure 3 shows that the increase in biomass (protein) was linear and stabilized at the last stage (pH 4) when the leaching rate sharply increased. Thus, we can assume that, in this case, the leaching increase was not determined by biomass growth directly.

We showed that pH 5.0 for acidophilic bacteria can be provided with the initial NAB inoculation without the additional input of sulfuric acid. These results show an approach to limit the input of sulfuric acid, however, they do not present a final solution of the whole industrial problem because NAB did not decrease pH lower than 5.0, while strong acidophilic bacteria such as *A. ferrooxidans* need a pH < 3.0.

Hydrometallurgical mining of valuable and non-ferrous metals by leaching is traditionally accompanied with acid effluent wastes. Pollution of mining territories with sulfuric acid is a global problem. The known attempts to replace strong sulfuric acid with organic acids and/or neutrophilic leaching bacteria, failed because of the weak leaching effect. In our experiments, we showed the intensification of nickel leaching by NAB *G. halophila* with bacterial stimulation. The formate-stimulated bioprocessing increased nickel production at pH 5.0–5.4. Pronk with coauthors [14,15] showed that formate is an additional source of energy for autotrophic bacteria. We suggest that, as well, it is a precursor of growth substrate (CO_2_ provided via formate degradation). As a whole, the experimental results showed that metal bioleaching can be intensified with the limited application of sulfuric acid.

## 4. Conclusions

Bench scale experiments showed that a high recovery of nickel from low-grade sulfide ores can be reached with stimulation of the leaching activity of neutrophilic bacteria *G. halophila* formate supplement. The leaching process was conducted within pH limits 7 → 5, which are favorable for environmental safety in nickel bioleaching. Nickel yield reached 69.75% of its content in the ore in 43 days. Increasing leaching activity may be explained by an increasing yield of bacteria, when the formate could serve both as a supplemented source of energy [14,15] and a precursor of growth substrate for autotrophic bacteria (CO_2_ provided via formate degradation). This approach is interesting both for increasing leaching efficiency and for reducing industrial sulfuric acid pollution.

## Figures and Tables

**Table 1:** Essential components of the low-grade sulfide nickel ore (CANMET-Mining and Mineral Sciences Laboratories).

Nos.	Minerals		Concentration, wt%
	Name	Composition	
1	pyrrhotite	Fe_(1−x)_S	33.0
2	plagioclase	(Na, Ca)AlSi_3_O_8_	22.0
3	hornblende	Ca_2_(Mg)O–2(Fe)_2_–4(Al, Fe)(Si_7_Al)O_22_(OH)_2_	13.0
4	hypersthene	(Mg, Fe)SiO_3_	5.0
5	pentlandite	(Fe, Ni)_9_S_8_	3.0
6	chalcopyrite	CuFeS_2_	0.7

**Table 2:** Activities of formate dehydrogenase in extracts of autotrophic bacteria.

Enzyme	Cofactors	Activity, nM/min·mg of Protein	
		*A. ferrooxidans* ATCC 21834 [14]	*G. halophila* VKM B-2757D
Formate dehydrogenase	PMS	80 (max.)	18
	NAD^+^	0	0

## Data Availability

Not applicable.
